# Gabapentin-Induced Priapism

**DOI:** 10.7759/cureus.21241

**Published:** 2022-01-14

**Authors:** Simon Kashfi, Justin Loloi, Iurii Statnii, Blerim Arifi, Shorabh Sharma

**Affiliations:** 1 Internal Medicine, CUNY School of Medicine, New York, USA; 2 Internal Medicine, Penn State Hershey Medical Center, Hershey, USA; 3 Internal Medicine, St. Barnabas Hospital Health System, Bronx, USA

**Keywords:** erection, ssri, non-ischemic priapism, ischemic priapism, gabapentin

## Abstract

Priapism is defined as an erection that lasts longer than four hours, is unrelated to sexual interest or stimulation, and is unrelieved by orgasm. The ischemic subtype is a urologic emergency and is often caused by medication side effects, most notably selective serotonin reuptake inhibitors and trazodone. We present the case of ischemic priapism thought to be caused by the recent initiation of gabapentin.

## Introduction

Priapism is defined as an erection that lasts longer than four hours, is unrelated to sexual interest or stimulation, and is unrelieved by orgasm [1-2]. The incidence of priapism in the general population is between 0.73 cases per 100,000 men per year [3]. Up to one-third (1/3rd) of cases of priapism are idiopathic [2]. Other etiologies include drug-induced, or related to, hematological abnormalities such as sickle cell disease. Trazodone and other antidepressants are the common culprits of drug-induced priapism. Priapism can be classified into three forms, ischemic (low flow), non-ischemic (high flow), and stuttering (also known as intermittent priapism) [1]. Ischemic priapism, which can be thought of as a compartment syndrome, is a urologic emergency that requires urgent management to preserve erectile function and prevent penile injury [4]. We present the case of a 54-year-old male with ischemic priapism thought to be caused by the recent use of gabapentin. To our knowledge, we describe the first reported case of gabapentin-induced priapism.

## Case presentation

A 54-year-old male with a history of hypertension, type 2 diabetes, hyperlipidemia, and depression presented to the emergency room 16 hours after the onset of a painful erection. He was sent from the hospital’s wound care clinic, which he goes to every two to three days for a diabetic foot ulcer. The erection was unprovoked and started while he was cooking. It was constant, painful, 10/10, and associated with dysuria. He denied penile discharge, scrotal pain, abdominal pain, fever, nausea, and back pain. The patient had been taking citalopram for eight months and had started taking gabapentin just 10 days prior to erection for the management of neuropathic foot pain. He denied any recent history of trauma to the penis. He denied any personal past or family history of priapism. He was not sexually active at the initiation of the erection or in the month prior. He did not smoke or use other illicit drugs (urine drug screen not collected).

On initial physical exam, the penile shaft was firm and the glans penis soft. The scrotum and perineum were non-tender, without erythema or edema. No inguinal lymphadenopathy was noted. Penile Doppler ultrasound was not performed, and scrotal ultrasound was negative for testicular torsion or an infectious process. Based on the history and physical exam, a diagnosis of ischemic priapism was made. Urology was consulted, and he subsequently underwent aspiration of the corpus cavernosum with the injection of phenylephrine. One-hundred twenty-five (125) cc of dark red blood was aspirated, and 1000 mcg of phenylephrine in 10 mL of normal saline was used. Corporal arterial blood gas was not obtained. The patient achieved detumescence and pain decreased to 5/10. He was admitted to the general medical floor for observation overnight.

The next morning, tumescence recurred with increasing pain. The patient was taken to the operating room for an urgent distal cavernosum-glans shunt, which resulted in satisfactory detumescence. On postoperation Day 1, the proximal part of the penis was soft, the distal part of the penis partially hard, and no tenderness was noted. The Foley catheter was removed, and the patient was cleared for discharge. However, early the next morning, the patient re-presented with erection, penile pain, and edema. He was taken to the operating room for a proximal penile shunt procedure, which again achieved detumescence. He was deemed stable and discharged on finasteride 5 mg daily for 90 days, and clopidogrel 75 mg daily for five days. Hypercoagulability and sickle-cell workup were negative (Table 1). It was determined that the priapism was caused by the recent introduction of gabapentin. The patient was discharged with citalopram and without gabapentin. He was called two months after discharge and denied other episodes of priapism while still taking the citalopram.

**Table 1:** Hypercoagulability Workup Normal values are in parenthesis. Hgb = Hemoglobin

Test	Result
Factor V Leiden Mutation	Negative
Protein C-Functional	135 (73-180%)
Protein S-Functional	102 (63-140%)
Antithrombin Antigen	123 (72-124%)
Hemoglobinopathy Fractionation Cascade	
Hgb A	97.8 (96.4-98.8 %)
Hgb A2	2.2 (1.8-3.2 %)
Hgb F	0.0 (0.0-2.0 %)
Hgb S	0.0 (0.0 %)

## Discussion

Ischemic priapism, otherwise known as low-flow priapism, occurs due to venous blockage that leads to a backup of blood and thus decreased arterial flow. This, in turn, causes hypoxia and penile pain. The cause is often idiopathic, but numerous drugs have been implicated in causing ischemic priapism [1,5]. In general, drugs can cause priapism by altering sympathetic pathways. For example, it is thought that antipsychotics cause priapism through an alpha-adrenergic blockade in the corpus cavernosum. Alpha-blockers such as prazosin can also cause priapism. Similarly, methylphenidates are thought to cause priapism through the inhibition of dopamine and norepinephrine reuptake [1]. It should be noted that, for at least prazosin, the relationship between dosage and priapism risk is unclear [6]. However, taking multiple doses of citalopram caused priapism in one case [7]. Treatment for ischemic priapism involves corporal blood aspiration followed by injection of alpha agonists directly into the corpus cavernosum. If this does not resolve the erection, shunt surgery can be performed. This allows the diversion of blood into another area of the penis. Generally, the distal shunt is performed first, followed by the proximal shunt in non-responders.

Non-ischemic priapism, otherwise known as high-flow priapism, is caused by penile trauma or iatrogenic injury, which can damage the cavernosal artery and create a fistula between the artery and the tissue. This type of priapism often presents two to three weeks after the initial injury. Because arterial blood is flowing, there is no hypoxia and no penile pain. Treatment is generally conservative, as the priapism often resolves on its own. If the erection persists, embolization of the fistula is performed. Stuttering priapism is characterized by recurrent episodes over the course of one’s life. It is a type of ischemic priapism and is most commonly associated with hematologic abnormalities such as sickle cell disease. Treatment is the same as with traditional ischemic priapism and is often augmented with preventative medications.

Our patient’s priapism was thought to be caused by gabapentin. The main rationale was that the priapism started just 10 days after starting the drug. This timing is similar to one case where a patient developed priapism about a month after starting paroxetine [8]. Additionally, it was our patient’s first-time taking gabapentin. That said, he was also taking citalopram, a selective serotonin reuptake inhibitor (SSRI), which is known to cause priapism [7]. However, he had been taking citalopram for eight months before his presentation and has continued to take it without issue. Interestingly, gabapentin is used to treat and prevent recurrent priapism because it has side effects of anorgasmia and decreased potency [4,9]. It is interesting to consider the possibility that the patient had priapism secondary to citalopram while on preventative treatment. However, we feel more strongly that it was, in fact, the gabapentin that triggered the episode. Our case is similar to one described by Hewett, who concluded that an episode of priapism was caused by trazodone even though the patient was also taking an SSRI [10]. It should also be noted that a case of priapism occurred with co-ingestion of olanzapine and gabapentin in a suicide attempt [11]. The authors concluded that the priapism was caused by the olanzapine, likely because the patient took 20 times his normal dose while only taking less than double his normal dose of gabapentin.

## Conclusions

Ischemic priapism is a urologic emergency often caused by medication side effects while non-ischemic priapism is caused by recent penile trauma. Providers should be aware of the etiology and management of different types of priapism should they evaluate a patient with a prolonged erection. We present a case of ischemic priapism thought to be caused by gabapentin use in a patient taking citalopram. This is unique because gabapentin is used for the prevention and treatment of stuttering priapism.
